# Transmissibility of influenza during the 21st-century epidemics, Spain, influenza seasons 2001/02 to 2017/18

**DOI:** 10.2807/1560-7917.ES.2020.25.21.1900364

**Published:** 2020-05-28

**Authors:** Lidia Redondo-Bravo, Concepción Delgado-Sanz, Jesús Oliva, Tomás Vega, Jose Lozano, Amparo Larrauri, the Spanish Influenza Sentinel Surveillance System

**Affiliations:** 1 Preventive Medicine Department, La Paz University Hospital, Madrid, Spain; 2 National Centre of Epidemiology, CIBER Epidemiología y Salud Pública (CIBERESP), Institute of Health Carlos III (ISCIII), Madrid, Spain; 3 Public Health Directorate, Castilla y León Regional Health Ministry, Valladolid, Spain; 4 The members of the network are listed at the end of this article

**Keywords:** influenza, surveillance, epidemiology, influenza-like illnesses

## Abstract

**Background:**

Understanding influenza seasonality is necessary for determining policies for influenza control.

**Aim:**

We characterised transmissibility during seasonal influenza epidemics, including one influenza pandemic, in Spain during the 21th century by using the moving epidemic method (MEM) to calculate intensity levels and estimate differences across seasons and age groups.

**Methods:**

We applied the MEM to Spanish Influenza Sentinel Surveillance System data from influenza seasons 2001/02 to 2017/18. A modified version of Goldstein’s proxy was used as an epidemiological-virological parameter. We calculated the average starting week and peak, the length of the epidemic period and the length from the starting week to the peak of the epidemic, by age group and according to seasonal virus circulation.

**Results:**

Individuals under 15 years of age presented higher transmissibility, especially in the 2009 influenza A(H1N1) pandemic. Seasons with dominance/co-dominance of influenza A(H3N2) virus presented high intensities in older adults. The 2004/05 influenza season showed the highest influenza-intensity level for all age groups. In 12 seasons, the epidemic started between week 50 and week 3. Epidemics started earlier in individuals under 15 years of age (−1.8 weeks; 95% confidence interval (CI):−2.8 to −0.7) than in those over 64 years when influenza B virus circulated as dominant/co-dominant. The average time from start to peak was 4.3 weeks (95% CI: 3.6–5.0) and the average epidemic length was 8.7 weeks (95% CI: 7.9–9.6).

**Conclusions:**

These findings provide evidence for intensity differences across seasons and age groups, and can be used guide public health actions to diminish influenza-related morbidity and mortality.

## Introduction

Influenza is an important cause of hospital admission and mortality during seasonal influenza epidemics, and as such, constitutes a major issue for public health, healthcare strategy and resource allocation. The latest estimates indicate that there are somewhere between 290,000 and 650,000 seasonal influenza-associated respiratory deaths each year worldwide, mostly affecting individuals over 64 years of age [1]. However, not all influenza epidemics have the same impact and epidemiological features. The impact of influenza on the health of the population depends on the epidemiology of the disease, population susceptibility by age and circulating viruses, as well as other contributing factors such as vaccine coverage, vaccine effectiveness, social mixing patterns, specific humidity and climate [2,3]. For this reason, analysing the start of the epidemic period, as well as the severity of the influenza epidemics every season, is important in order to be able to alert health services and consequently ameliorate the morbidity, mortality and economic costs associated with influenza.

Three different indicators have been proposed to assess influenza epidemic/pandemic severity: transmissibility of the virus, clinical severity caused by the infection, and impact on the healthcare sector and on society [4]. Transmissibility, the indicator we focus on in this study, indicates the ease with which a virus may spread person-to-person and is therefore an important determinant of the number of people who will be affected. This can be measured using influenza incidence data obtained through routine influenza surveillance systems. The Spanish Influenza Sentinel Surveillance System (SISSS) provides weekly influenza-like illness (ILI) incidence and percentage of positive ILI samples for influenza, which enables the identification of the start of the epidemics and the calculation of influenza activity levels. This information is crucial to helping guide decisions about public health interventions that can potentially reduce the impact of an influenza epidemic.

The moving epidemic method (MEM) has been adopted by the European Centre for Disease Prevention and Control (ECDC) and the World Health Organization Regional Office for Europe (WHO/Europe) to standardise approaches across Europe for calculating epidemic thresholds and intensities. Most countries in the WHO European Region are already using this method for weekly influenza reports [5]. In Spain, it has been used at both regional and national level since 2015 after an agreement between all of the autonomous regions was reached [6]. Consolidating the use of this methodology with a systematic assessment of the transmissibility of the influenza epidemics has also been piloted and implemented in different countries around the world with the same purpose [7-10].

In our study, we focused on characterising the transmissibility during seasonal influenza epidemics, including the 2009 influenza A(H1N1) pandemic, in Spain for the 2001/02 to 2017/18 influenza seasons using the MEM [5,11] and an epidemiological-virological parameter (Goldstein’s proxy), in order to calculate intensity thresholds and intensity levels of the influenza epidemics that have occurred in Spain during the 21st century.

This study forms part of an ongoing investigation that includes other analysis using seriousness of disease and impact, the two other indicators proposed, to complete an assessment of seasonal and pandemic influenza severity [4].

## Methods

### Data source and influenza surveillance parameters

The SISSS has been described in detail in previous articles [12]. However, as a brief overview, sentinel physicians, general practitioners and paediatricians, voluntarily report cases of influenza-like illness (ILI) on a weekly basis, following a definition based on the European Union’s (EU) ILI case definition [13]. They systematically swab, nasal or nasopharyngeal, the first two ILI patients consulting each week and send the swabs to network-affiliated laboratories for influenza virus detection. For the influenza seasons 2001/02 to 2017/18, we used data obtained from the SISSS to estimate a modified version of Goldstein’s proxy for influenza incidence, hereafter referred to as Proxy [14], defined as the weekly age-specific ILI rate (ILI cases/100,000 population) multiplied by the all-ages weekly percentage of samples that test positive for influenza, for all ages and by age group (< 15, 15–64 and > 64 years of age).

### Moving epidemic method intensity thresholds and parameters

We calculated the epidemic threshold (ET) and intensity thresholds (ITs) applying the MEM [5,11] to the weekly proxy of influenza incidence from the 2001/02 to 2017/18 influenza seasons, excluding the 2009/10 pandemic season because of the different dynamic of the pandemic [15]. The ET was calculated as the upper limit of the 95% one-sided confidence interval (CI) of the arithmetic mean of the 30 highest pre/post-epidemic weekly rates. The three ITs were calculated as the upper limit of the 40% (IT40), 90% (IT90) and 97.5% (IT97.5) one-sided CIs of the geometric mean of the 30 highest weekly epidemic rates [5,11]. These four cut-off points defined five intensity levels: baseline (below epidemic threshold), low, medium, high and very high intensity.

According to the ET, we calculated the starting week of the epidemic period as first week above the ET, and the length of the epidemic period as the number of weeks above the ET. To identify possible differences in the influenza intensity levels, we also applied MEM by age group (< 15, 15–64 and > 64 years of age).

We also explored the differences among starting weeks and peak weeks, across age groups by dominant/co-dominant virus. Dominance was defined as a virus circulating during a season at a proportion ≥ 60% and co-dominance when circulating at a proportion > 40% and < 60%.

Finally, we calculated the average time (weeks) from the starting week to the peak and the length of the epidemic period (weeks), according to seasonal virus circulation, and established potential differences between dominant virus or mixed circulation by using an analysis of variance (ANOVA) or Student’s t-test, as appropriate, and the corresponding 95% CI. A p value < 0.05 was considered statistically significant.

All analyses were conducted using R software version 3.4.0 (R Foundation, Vienna, Austria), and the package *mem* version 2.14 [15], the official implementation of MEM.

## Results

### Moving epidemic method characterisation of influenza epidemics’ intensities

**Figure 1:**
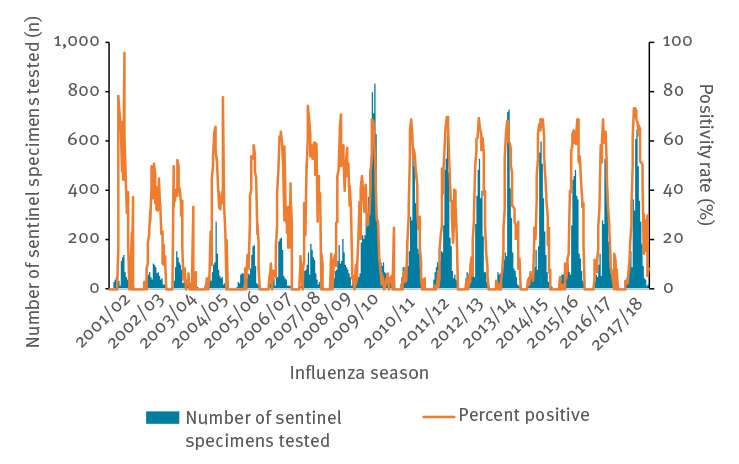
Weekly analysed swabs and influenza positivity rate, Spain, influenza seasons 2001/02–2017/18

The characteristics of the SISSS and the surveillance information from 2001/02 to 2017/18 are presented in Table 1. The number of sentinel specimens tested is presented with the percentage of positivity rate in Figure 1. An increase in the number of specimens tested from 2009 was observed, since systematic swabbing was introduced in the SISSS during the 2009/10 pandemic season.

**Table 1:** Characteristics of the Spanish Influenza Sentinel Surveillance System (SISSS) and surveillance information, Spain, influenza seasons 2001/02–2017/18

Influenza season	Number of sentinel physicians^a^	Surveilled population (n)	Percentage of surveilled population^b^ (%)	ILIs notified (n)	Sentinel specimens tested (n)	Positivity rate (%)	Dominant/co-dominant virus^c^
2001/02	342	320,102	0.8	8,488	1,214	43.4	A(H3N2)
2002/03	340	318,736	0.8	4,684	1,437	30.5	B
2003/04	422	395,137	0.9	8,045	1,410	30.9	A(H3N2)
2004/05	453	568,906	1.3	18,223	1,781	46.0	A(H3N2)
2005/06	538	648,676	1.5	9,082	1,885	30.6	A(H1N1)/B
2006/07	668	619,830	1.4	12,381	1,848	45.1	A(H3N2)
2007/08	646	612,619	1.4	12,732	2,023	49.6	A(H1N1)/B
2008/09	698	667,414	1.5	12,688	2,538	42.5	A(H3N2)
2009/10	867	842,190	1.9	24,459	11,218	41.7	A(H1N1)pdm09
2010/11	841	820,277	1.8	17,224	5,482	45.0	A(H1N1)pdm09
2011/12	887	845,380	1.8	18,873	5,858	50.1	A(H3N2)
2012/13	831	819,328	1.8	17,876	5,173	51.5	B
2013/14	873	832,189	1.8	15,864	5,060	50.3	A(H1N1)pdm09/A(H3N2)
2014/15	788	765,065	1.6	18,955	5,101	54.5	A(H3N2)
2015/16	823	796,472	1.7	16,951	5,308	51.1	A(H1N1)pdm09
2016/17	789	779,536	1.7	13,430	4,478	47.8	A(H3N2)
2017/18	797	800,016	1.7	19,505	6,034	58.2	B/A(H3N2)

ILI: influenza-like illness.

^a^ General practitioners and paediatricians.

^b^ Of the total population of the Spanish regions participating in the SISSS each influenza season.

^c^ A virus that circulated in a proportion ≥ 60% was considered dominant, and co-dominant in a proportion > 40 and < 60%.

**Figure 2:**
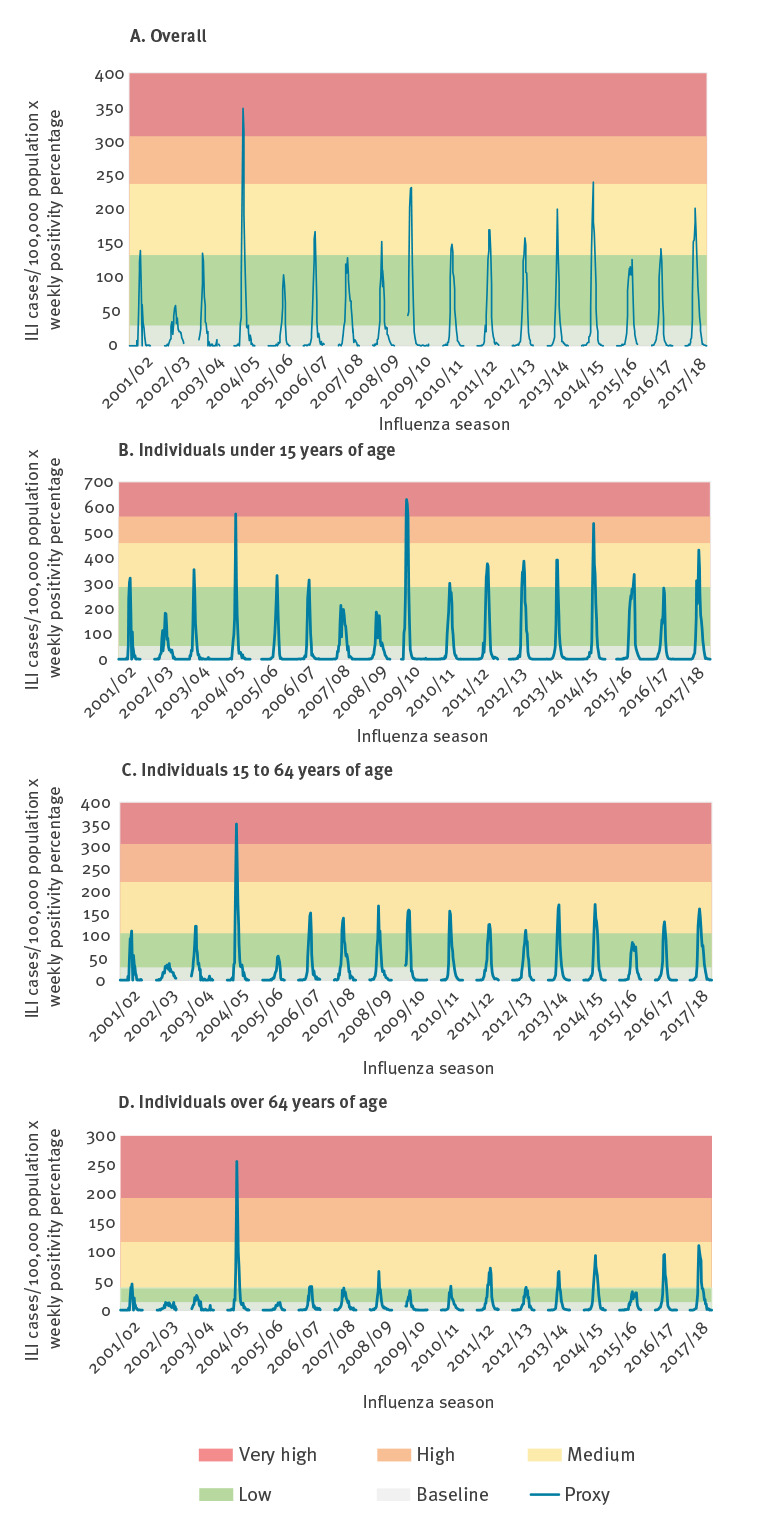
Influenza epidemic thresholds and intensity levels according to the moving epidemic method (MEM), overall (A) and by age group (B,C,D), using Proxy^a^, Spain, influenza seasons 2001/02–2017/18

^a^ Proxy is defined as weekly age-specific influenza-like illness (ILI) cases/100,000 population multiplied by the all-ages weekly percentage of samples that test positive for influenza.

Influenza epidemics’ intensity levels for the 2001/02 to 2017/18 influenza seasons were characterised based on the epidemic and intensity thresholds (Figure 2). Fourteen seasons showed a peak with low or medium intensity levels, one season reached a high level (2014/15) and one season presented a very high peak intensity level (2004/05) (Figure 2 and Table 2).

**Table 2:** Influenza epidemics by dominant/co-dominant virus, starting week, peak week, length and peak intensity according to the moving epidemic method (MEM) using Proxy^a,^ Spain, influenza seasons 2001/02–2017/18

Influenza season	Dominant/co-dominant virus^b^	Starting week	Peak week	Epidemic length (weeks)	Peak intensity^c^
2001/02	A(H3N2)	1	4	10	Low
2002/03	B	51	4	11	Low
2003/04	A(H3N2)	43	47	9	Low
2004/05	A(H3N2)	50	2	12	Very high
2005/06	A(H1N1)/B	8	11	7	Low
2006/07	A(H3N2)	2	6	8	Medium
2007/08	A(H1N1)/B	48	2	14	Low
2008/09	A(H3N2)	50	1	9	Medium
2009/10	A(H1N1)pdm09	40	46	11	Medium
2010/11	A(H1N1)pdm09	51	2	10	Medium
2011/12	A(H3N2)	52	7	11	Medium
2012/13	B	3	8	10	Medium
2013/14	A(H1N1)pdm09/A(H3N2)	1	4	8	Medium
2014/15	A(H3N2)	2	5	10	High
2015/16	A(H1N1)pdm09	4	9	10	Low
2016/17	A(H3N2)	50	3	9	Medium
2017/18	B/A(H3N2)	50	3	13	Medium

^a^ Proxy is defined as weekly age-specific influenza-like illness (ILI) cases/100,000 population multiplied by the all-ages weekly percentage of samples that test positive for influenza.

^b^ A virus that circulated in a proportion ≥ 60% was considered dominant, and co-dominant in a proportion> 40 and < 60%.

^c^ Level of intensity reached in the week with maximum influenza activity of the season.

Influenza A(H3N2) has been the virus circulating as dominant/co-dominant most often (10 seasons) and in one of the seasons, the intensity reached was very high, in one it was high, in six it was medium and in two it was low. When influenza A(H1N1)/A(H1N1)pdm09 virus was dominant/co-dominant (six seasons), the intensity reached was medium in three and low in three. Finally, when influenza B virus was the dominant/co-dominant virus (five seasons), the intensity reached was low in three and medium in two (Table 2).

ET and IT were higher for individuals under 15 years of age than for the 15 to 64, and over 64 years of age groups (Figure 2 and Table 3). In the under 15 years of age group, two seasons (2004/05 and 2009/10) reached a very high intensity level, and one reached a high level (2014/15). For the 15 to 64 and over 64 years of age groups, the intensity was very high only in 2004/05; the rest of the seasons presented a medium or low intensity level (Figure 2).

**Table 3:** Influenza epidemic thresholds and intensity thresholds according to the moving epidemic method (MEM) using Proxy^a^, Spain, influenza seasons 2001/02–2017/18

Age group (years)	Epidemic thresholds	Intensity thresholds		
		Low	Medium	High
<15	52.4	285.3	457.3	563.4
15–64	29.0	105.3	220.6	305.8
> 64	13.94	38.1	117.4	193.0
All ages	29.3	132.3	237.1	306.8

^a^ Proxy is defined as weekly age-specific influenza-like illness (ILI) cases/100,000 population multiplied by the all-ages weekly percentage of samples that test positive for influenza.

### Epidemic start and total length assessment

We also used MEM with Proxy to study the influenza epidemics’ start and peak weeks, by age group. From 2001/02 until 2017/18, besides the 2009/10 pandemic which started in week 40, the earliest epidemic started in week 48 (2007/08), and the latest in week 8 (2005/06). In 12 seasons, the epidemic started between week 50 and week 3. By age group, we estimated that during the study period, influenza epidemics started earlier in those under 15 years of age (–1.8 weeks; CI 95%: –2.8 to –0.7) than in those over 64 years of age, when B virus circulated as dominant/co-dominant considering the virus circulation within the entire season. There were not significant differences regarding the starting week when compared with the 15 to 64 years of age group (Table 4). Influenza epidemics did not peak earlier in individuals under 15 years than for those 15 to 64 or those over 64 years of age, regardless of the virus type/subtype (Table 4).

**Table 4:** Differences in starting and peak weeks by age group and dominant/co-dominant influenza virus, using Proxy^a^, Spain, influenza seasons 2001/02–2017/18

Differences	Age groups compared (years)	Dominant/co-dominant virus^b^	Mean	95% CI	
				Lower	Upper
Among starting weeks	< 15 vs 15–64	A(H1N1)^c^	−0.2	−1.2	0.8
		A(H3N2)	0.0	−0.8	0.8
		B	−0.8	−2.3	0.8
	< 15 vs > 64	A(H1N1)^c^	−0.6	−2.8	1.6
		A(H3N2)	0.0	−0.8	0.8
		B	−1.8	−2.8	−0.7
Among peak weeks^d^	< 15 vs 15–64	A(H1N1)^c^	0.0	−1.5	1.5
		A(H3N2)	0.0	−0.7	0.7
		B	−1.3	−3.1	0.6
	< 15 vs > 64	A(H1N1)^c^	0.8	−1.7	3.3
		A(H3N2)	−0.3	−1.0	0.5
		B	0.5	−2.0	3.0

CI: confidence interval.

^a^ Proxy is defined as weekly age-specific influenza-like illness (ILI) cases/100,000 population multiplied by the all-ages weekly percentage of samples that test positive for influenza.

^b^ A virus that circulated within the season in a proportion ≥ 60% was considered dominant and co-dominant in a proportion > 40 and > 60%.

^c^ Influenza A(H1N1)pdm09 and A(H1N1) virus.

^d^ Influenza season 2015/16 was excluded from this analysis as there was not a clear peak week; a plateau was observed between weeks 7 and 12.

In a sensitivity analysis, considering the dominant/co-dominant virus during the starting weeks of the epidemic, we obtained differences in the timing of the peak week in individuals under 15 years vs those over 64 years of age (–2.3 weeks; 95% CI: –3.3 to –1.3) when B circulated as dominant/co-dominant (data not shown).

We also examined the time from start to peak and total epidemic length according to the dominant/co-dominant influenza virus. Overall, across the 17 influenza seasons studied, the average time from start to peak was 4.3 weeks and the average total epidemic length was almost 9 weeks (Table 5). No differences were found in the number of weeks from start to peak (p = 0.47) according to the dominant virus. The length of the epidemic was longer for seasons with a predominance influenza B virus (10.5 weeks) than those seasons with a predominance of influenza A(H1N1)pdm09 or A(H3N2) virus (8.0 and 8.3 weeks, respectively); these differences were statistically significant. We also assessed these two parameters in seasons with circulating virus co-dominance vs seasons with only one dominant virus and found no significant differences (Table 5).

**Table 5:** Average weeks from start to peak week, and influenza epidemic length by dominant/co-dominant virus, using Proxy^a^, Spain, influenza seasons 2001/02–2017/18

Influenza virus circulation		Average time from start to peak (weeks)				Epidemic length (weeks)			
		Mean	95% CI		p value	Mean	95% CI		p value
			Lower	Upper			Lower	Upper	
Dominant/co-dominant virus^b^	A(H1N1)^c^	4.4	2.3	6.5	0.47	8.0	6.5	9.5	0.03
	A(H3N2)	3.9	3.0	4.7		8.3	7.5	9.0	
	B	5.0	4.1	5.9		10.5	8.7	12.3	
Dominance/co-dominance season^d^	Dominance seasons	4.3	3.4	5.2	0.96	8.4	7.9	9.0	0.13
	Co-dominance seasons	4.3	2.9	5.8		10.0	5.8	14.2	
Overall		4.3	3.6	5.0	NA	8.7	7.9	9.6	NA

CI: confidence interval; NA: not applicable.

^a^ Proxy is defined as the weekly age-specific influenza-like illness (ILI) cases/100,000 population multiplied by the all-ages weekly percentage of samples that test positive for influenza.

^b^ A type of virus that circulated in a proportion ≥ 60% was considered dominant, and co-dominant in a proportion > 40 and  <60%. Within influenza A virus, the same rule is applied to dominant/co-dominant subtype.

^c^ Influenza A(H1N1)pdm09 and A(H1N1) virus.

^d^ Dominance season is when a dominant influenza virus has been circulating and co-dominance season is when a type/subtype co-dominant virus has been circulating.

## Discussion

This study characterises the transmissibility of the first 17 seasonal influenza epidemics, including the 2009 influenza A(H1N1) pandemic, that have occurred in Spain since 2001/02. We used the MEM to define influenza-intensity levels and to study the differences in the temporal presentation of influenza epidemics. Using a parameter that includes both epidemiological and virological information (Proxy), individuals under 15 years of age presented higher transmissibility than older age groups. Overall, influenza epidemics started earlier in individuals under 15 years of age than in the those over 64 years of age when influenza B was the dominant/co-dominant virus. Most influenza seasons started between mid-December and the third week of January, the average from start to peak was just over 4 weeks and the average total epidemic length almost 9 weeks.

We evaluated the transmissibility of the 17 influenza epidemics with the MEM. This is a robust tool to calculate the epidemic threshold and identify the week when influenza epidemics start; this is key to helping public health authorities plan and organise healthcare resources during the influenza season. We used the MEM because it determines the levels of influenza activity mathematically rather than using a qualitative assessment, compared with previous qualitative EISN (European Influenza Surveillance Network) intensity indicators, thereby avoiding frequent inconsistencies in surveillance reporting. Compared with other existing methods, the advantages of MEM are its simplicity, flexibility and intuitiveness. Other methods aiming to detect the start of influenza activity rely on relatively complex mathematical models [16-19] that require specialist programs which limits their implementation. The MEM method is developed on open-source software which makes its implementation user-friendly. However, because it requires a minimum of 5 years of consistent historical influenza data to accurately calculate values, similar analyses may not be possible in countries where these historical data are not available. In this study we calculated common MEM thresholds, to retrospectively analyse the entire study period and compare the intensity levels by age group and the inter-season variability of transmissibility. For routine surveillance purposes, we use a MEM approach in which the on-going influenza season is compared with recent seasons through a moving period of 10 previous influenza epidemics length. Even though including more than 10 seasons can potentially increase accuracy, it can also make the model more susceptible to be affected by secular trends [5]. Season-specific thresholds might produce some controversial intensity levels, because such characterisation closely depends on recent historical influenza data. For example, seasons of extremely high intensity might impact on the model and the highest intensity thresholds, what will allow characterising the following high intensity epidemics within lower thresholds. This fully supports the idea of assessing the intensity of an influenza epidemic, in terms of comparison with the intensity of previous influenza seasons.

In our study, we used a modified version Goldstein’s proxy for as parameter to calculate the epidemics’ characteristics and intensity thresholds. The EU ILI case definition is based on signs and symptoms, which cannot be distinguished from other respiratory illnesses with similar clinical presentations, especially respiratory syncytial virus (RSV) infection in children. To make the proxy more accurate though, we applied the influenza positivity rate to ILI rates and consequently accounted for confirmed influenza cases (Proxy). In addition, by using the amended Proxy we avoided the disadvantage of purely using a percentage of virologically positive specimens, especially at the start and the end of the epidemic when low sample sizes inherently unstable may bias results. This was the rationale behind the use of Proxy in this study, in accordance with other authors who have used positivity data to assess influenza epidemics’ intensity [20]. Goldstein’s proxy has also been used in different models to calculate other indicators of influenza severity such as influenza-attributable mortality [4].

Having information on influenza intensity by age group is key to ensuring optimal resource allocation and healthcare setting management [21,22]. Our results showed higher influenza transmissibility for individuals under 15 years of age during the 2009 influenza A(H1N1) pandemic, which is similar to the transmission levels already described by other authors [23,24]. This group was responsible for the high overall transmissibility detected during the pandemic. Individuals under 15 years of age were also heavily affected in the 2004/05 influenza season, when an influenza A(H3N2) virus drift variant circulated [7]. Notably, we found increased incidence rates in all age groups during this season. Individuals under 15 years of age were also heavily affected during the A(H3N2) virus-dominant 2014/15 influenza season. This season had a reportedly low in-season vaccine effectiveness against A(H3N2), which is in line with the substantial impact of the 2014/15 influenza epidemic in Spain on population mortality [25]. Influenza seasons 2016/17 and 2017/18, which had dominance/co-dominance of A(H3N2) virus, but with a medium intensity overall, presented higher epidemic intensities in those over 64 years of age than in previous seasons. These differences may be explained by the interaction of several factors, including vaccine coverage, vaccine effectiveness, antigenic changes and immunological memory [26-28].

Some authors have proposed the possibility of children acting as drivers of influenza epidemics [29,30] or as playing a primary role in intra-household transmission [31], with their role in influenza B outbreaks being highlighted [32,33]. However, there is not yet broad agreement on this issue [29]. In the present study, we detected a delay of ca 2 weeks in individuals over 64 years of age when compared with those under 15 years of age when the circulating virus was influenza B. This would support children being drivers of epidemics when influenza B virus is predominant. It has been noted by other authors that early starting of influenza activity in children could also be influenced by the presence of other circulating viruses, such as RSV and parainfluenza, which can affect children considerably [34,35]. However, by using Goldstein’s proxy we should have largely avoided the influence of other viruses on the results. It could also be argued that the earlier influenza activity start in children might be related to different health seeking behaviour, with children consulting earlier after symptom onset than other age groups, not only because of clinical factors, but also with a parent’s risk-perception after seeing information in the media in a specific season. However, that this earlier epidemic start in individuals under 15 years of age is only detected when influenza B is the dominant/co-dominant virus, and not when A viruses are dominant/co-dominant, supports the notion of individuals under 15 years of age being drivers of influenza B epidemics. In contrast, we found no differences in the peak week by type/subtype of the dominant virus among age groups. These data suggest that although children have an earlier presentation than adults when B viruses circulate, the highest activity is reached at the same time in both groups. This may reflect slower or interrupted transmission among children, being explained by the intervention of the Christmas holiday period, which would halt school-associated transmission and slow the progression of the epidemic [36].

We observed an average of 4 weeks between the start of the epidemic and its peak; this is in accordance with a recently published study carried out in Scotland using primary care virological data. This finding demonstrates that MEM is a robust tool, not only to set intensity thresholds but also to predict the weeks of maximum intensity once the epidemic baseline has been exceeded [9].

Regarding the total length of the epidemics, those influenza seasons with B virus dominance/co-dominance lasted ca 2 weeks longer than those with A viruses. We believe that this can probably be explained by the earlier start in children. Overall, once the epidemic threshold is crossed, healthcare services can expect an average of 9 weeks of influenza-related demands.

Taking the differences in intensity regarding the dominant/co-dominant virus circulating into account, we observed that influenza B epidemics present lower intensity but last longer. This means that the amount of resources allocated in the higher demand of medical attention could be smaller but during a more prolonged period. On the contrary, during influenza A epidemics, the probability of reaching a medium (during A(H1N1)pdm09 epidemics) or high (during A(H3N2) epidemics) level of intensity is higher but the epidemic length is shorter. For this reason, the amount of resources and their organisation must be envisaged to diminish the effects of influenza epidemics considering the dominant/co-dominant virus circulating.

A main limitation of our study is that changes were introduced within the SISSS over the seasons. One of most important was the introduction of systematic swabbing in the SISSS during the 2009/10 pandemic season (Figure 1) [37], which made the age-positivity rate much closer to the real situation. Before the 2009/10 pandemic, there was a tendency to collect fewer specimens than recommended as the age of patients increased, with this especially being the case for patients 65 years of age and above [38]. However, after systematic swabbing was introduced, the frequency of swabbing became more homogeneous among all age groups [39]. Thus, the data should before 2009 should be considered less representative while that from 2009 onwards should be considered more representative of the surveyed population. Another limitation is that there was a change in the ILI case definition between the influenza season 2008/09 and the 2009/10 pandemic, from the International Classification of Health Problems in Primary Care (ICHPPC-2) definition [40] to the EU ILI case definition [13]. Although there is no direct comparison between these two definitions, the EU ILI case definition has been reported to have higher sensitivity than the WHO and the US Centers for Disease Control and Prevention (CDC) case definition [41]. It is thus possible that it performs different than the definition of the ICHPPC which could influence the intensity level of the epidemics after the 2009/10 season. However, because we calculated the MEM IT with the values of all epidemics from 2001/02 to 2017/18, the possible differences in such a characterisation probably decreases. Nevertheless, the SISSS has demonstrated considerable flexibility in order to incorporate changes in the dynamic and methods of surveillance [42].

We consider that the main strength of our study to be the long time period and number of influenza seasons with epidemiological and virological data included in series, overall and by age group, obtained from a well-established influenza sentinel surveillance system [6,33].

## Conclusion

 Applying our method as part of weekly influenza surveillance during subsequent influenza seasons and possible pandemics will allow for the assessment of when influenza activity will start in real-time. This will further allow to predict when there will be maximum pressure on the healthcare system so as to guide public health measures and resource planning in a way that may reduce the impact of the morbidity and mortality associated with seasonal and pandemic influenza. We believe that the approach shown might be very useful to any European country to identify the starting of a seasonal influenza epidemic. In addition, we are able to inform on the level of influenza transmissibility, and therefore how easily the epidemic will spread among various population groups. Those seasons with a considerable circulation of B virus would warrant a closer monitoring of infection in children because the disease would likely be starting within this population group.

## Members of the Spanish Influenza Surveillance System not already named as an author at the beginning of the manuscript

Andalucía: Virtudes Gallardo (Servicio de Epidemiología, Consejería de Salud, Sevilla); Jose María Navarro (Hospital Virgen de las Nieves de Granada). Aragón: Miriam García (Servicio de Vigilancia en Salud Pública, Dirección General de Salud Pública, Zaragoza); Ana María Martínez (Hospital Universitario Miguel Servet, Zaragoza). Asturias: Ismael Huertas (Dirección General de Salud Pública, Consejería de Salud , Oviedo); Santiago Melón (Hospital Universitario Central de Asturias de Oviedo). Baleares: Jaume Giménez, Juana María Vanrell (Servicio de Epidemiología, Dirección General de Salud Pública, Palma de Mallorca); Jordi Reina (Hospital Son Espases de Palma de Mallorca). Canarias: Nieves López Gonzalez-Coviella (Servicio de Epidemiología y Prevención, Consejería de Sanidad); Carmen Pérez (Hospital UGGC Dr Negrín de Las Palmas de Gran Canarias). Cantabria: Luis Viloria (Sección de Epidemiología, Consejería de Sanidad-Gobierno de Cantabria, Santander); Mónica Gozalo (Hospital Universitario Marqués de Valdecilla-IDIVAL, Santander). Castilla la Mancha: Sonia Humanes, María Victoria García (Servicio de Epidemiología, Dirección General de Salud Pública, Toledo). Castilla y León: Raúl Ortiz de Lejarazu (Hospital Clínico Universitario de Valladolid, WHO- National Influenza Centre). Cataluña: Nuria Torner (Subdirecció General de Vigilància i Resposta a Emergències en Salut Pública, Agència de Salut Pública, CIBERESP, Barcelona); María de los Ángeles Marcos (Hospital Clínic de Barcelona, WHO- National Influenza Centre). Comunitat Valenciana: Aurora López (Subdirección General de Epidemiologia y Vigilancia de la Salud, Conselleria de Sanitat, Valencia); Concepción Gimeno (Consorci Hospital General Universitari de València). Extremadura: Julián Mauro Ramos (Subdirección de Epidemiología. Dirección General de Salud Pública, Servicio Extremeño de Salud, Mérida); Guadalupe Rodríguez (Complejo Hospitalario San Pedro de Alcántara, Cáceres). Madrid: Luis García (Servicio de Epidemiología. Dirección General de Salud Pública, Madrid); Juan Carlos Galán (Hospital Universitario Ramón y Cajal, Madrid); Lola Folgueira (Hospital Universitario Doce de Octubre, Madrid). Navarra: Jesús Castilla (Sección de Vigilancia de Enfermedades Transmisibles del Instituto de Salud Pública, CIBERESP, Pamplona); Mirian Fernández-Alonso (Clínica Universidad de Navarra, Pamplona); Carmen Ezpeleta (Complejo Hospitalario de Navarra, Navarra). País Vasco: Fernando González Carril (Servicio de Vigilancia Epidemiológica y Vacunas, Dirección de Salud Pública y Adicciones, Vitoria-Gasteiz); Gustavo Cilla (Hospital Universitario Donostia de San Sebastián, CIBERES). La Rioja: Carmen Quiñones (Servicio de Epidemiología y Prevención Sanitaria, Dirección General de Salud Pública y Consumo); Miriam Blasco (Hospital San Pedro de Logroño). Ceuta: Ana Rivas (Sección de Vigilancia Epidemiológica, Consejería de Sanidad y Bienestar Social); José López (Hospital Universitario de INGESA). Melilla: Daniel Castrillejo (Servicio de Epidemiología, Dirección General de Sanidad y Consumo, Consejería de Presidencia y Salud Pública). National Centre for Microbiology, National Influenza Reference Laboratory, WHO-National Influenza Centre, ISCIII, Majadahonda: Francisco Pozo, Inmaculada Casas.
